# Lycopene Modulates Pathophysiological Processes of Non-Alcoholic Fatty Liver Disease in Obese Rats

**DOI:** 10.3390/antiox8080276

**Published:** 2019-08-05

**Authors:** Mariane Róvero Costa, Jéssica Leite Garcia, Carol Cristina Vágula de Almeida Silva, Artur Junio Togneri Ferron, Fabiane Valentini Francisqueti-Ferron, Fabiana Kurokawa Hasimoto, Cristina Schmitt Gregolin, Dijon Henrique Salomé de Campos, Cleverton Roberto de Andrade, Ana Lúcia dos Anjos Ferreira, Camila Renata Corrêa, Fernando Moreto

**Affiliations:** 1 Medical School, São Paulo State University (Unesp), Botucatu 18618-687, Brazil; 2 School of Dentistry, São Paulo State University (Unesp), Araraquara 14800-901, Brazil

**Keywords:** metabolic syndrome, NAFLD, hypercaloric diet, obesity

## Abstract

*Background*: The higher consumption of fat and sugar are associated with obesity development and its related diseases such as non-alcoholic fatty liver disease (NAFLD). Lycopene is an antioxidant whose protective potential on fatty liver degeneration has been investigated. The aim of this study was to present the therapeutic effects of lycopene on NAFLD related to the obesity induced by a hypercaloric diet. *Methods*: Wistar rats were distributed in two groups: Control (Co, *n* = 12) and hypercaloric (Ob, *n* = 12). After 20 weeks, the animals were redistributed into the control group (Co, *n* = 6), control group supplemented with lycopene (Co+Ly, *n* = 6), obese group (Ob, *n* = 6), and obese group supplemented with lycopene (Ob+Ly, *n* = 6). Ob groups also received water + sucrose (25%). Animals received lycopene solution (10 mg/kg/day) or vehicle (corn oil) via gavage for 10 weeks. *Results*: Animals which consumed the hypercaloric diet had higher adiposity index, increased fasting blood glucose, hepatic and blood triglycerides, and also presented in the liver macro and microvesicular steatosis, besides elevated levels of tumor necrosis factor-α (TNF-α). Lycopene has shown therapeutic effects on blood and hepatic lipids, increased high-density lipoprotein cholesterol (HDL), mitigated TNF-α, and malondialdehyde (MDA) and further improved the hepatic antioxidant capacity. *Conclusion*: Lycopene shows therapeutic potential to NAFLD.

## 1. Introduction

Obesity is a chronic disease with high prevalence worldwide, which represents an overwhelming problem for public health [1,2]. Recent estimates indicate that more than 1.9 billion adults are overweight and of these, more than 650 million are obese [3]. Although weight control is a consequence of a complex interaction between genetic, metabolic, psychological, and social factors, the obese phenotype is broadly influenced by behavioral aspects [4,5]. The energy imbalance resulting from the hypercaloric foods consumption and sedentary lifestyle corroborates for such an outcome [6]. The higher intake of processed foods containing saturated fat, added sugar, and high energy density led to an increase in the caloric value of the diet [7]. Taken together these factors favor the development of obesity and its related diseases such as nonalcoholic fatty liver disease (NAFLD) [8,9].

NAFLD comprises a spectrum of diseases ranging from simple steatosis to biochemical and functional abnormalities in the liver, which may result in an inflammatory process known as nonalcoholic steatohepatitis (NASH). If this problem is not solved, it may progress to hepatic cirrhosis and hepatocellular carcinoma [9]. It is hypothesized that these liver changes are the result of a two-step process, named the “two hit” theory. The first hit results from the imbalance between hepatic lipid influx and hepatic lipid clearance. The second hit involves the inflammatory and oxidative process [10,11]. Another hypothesis attributed to the NAFLD genesis is the “multiple parallel-hit”, which comprises several parallel hits including insulin resistance, inflammation, oxidative stress, genetic and epigenetic mechanisms, environmental elements, and microbiota changes [11]. Except for some divergences, these hypotheses present common mechanisms, including oxidative stress and inflammation, which play a central role in the genesis of NAFLD.

Dietary factors are also important to liver fat deposition [10]. The excess of nutrients received by the liver are converted into triglycerides, which can be oxidized or exported to extra-hepatic organs [12,13]. In the presence of huge amounts of the substrate, the mitochondria increase the production of reactive oxygen species (ROS), impairing its function [14]. ROS are capable of activating inflammatory pathways and the inflammatory cells increase the ROS production, which leads to tissue damage [15]. Moreover, components of the diet itself can directly lead to the activation of inflammatory pathways, such as saturated fatty acids [16], or also lead to intestinal dysbiosis and consequent infiltration of lipolysaccharides into the bloodstream, contributing to the inflammatory process [17]. Considering this overview and the pathological consequences of NAFLD, it is important to seek effective alternatives to treat or prevent hepatic accumulation of lipids and its unfolding. A wide range of drugs are available for the treatment of NAFLD in order to avoid its progress, including the use of antioxidants, such as lycopene [18].

Lycopene is a carotenoid without provitamin A activity, which gives red colors to vegetables such as tomatoes, red grapefruit, watermelon, and apricots [19]. This carotenoid is a powerful antioxidant with anti-inflammatory activity [20], which has been shown to modulate important metabolic processes in the body, attenuating complications of obesity and restoring liver function. The lycopene is a liposoluble molecule, which depends on dietary lipids to be absorbed. The liver is one of the major organs that stores lycopene, so in this point of view, the investigation of lycopene effects on NAFLD is valuable [19]. Thus, the aim of this study is to verify lycopene’s therapeutic potential on the pathophysiological processes of NAFLD in obese animals fed a hypercaloric diet.

## 2. Materials and Methods

### 2.1. Animals and Experimental Protocol

This studied was approved by the Ethics Committee on the Use of Animals (CEUA protocol number 1266/2018) of Botucatu Medical School, Sao Paulo State University. The experiment was performed in accordance with the National Institute of Health’s Guide to the Care and Use of Laboratory Animals [21]. Young male Wistar rats (8 weeks old) were kept in a controlled environment of temperature (22 ± 3 °C), luminosity (12 h light-dark cycle), and humidity (relative humidity of 60 ± 5%). Initially, the animals were distributed in two experimental groups fed different diets for induction of obesity: Control diet (*n* = 12) and hypercaloric diet (*n* = 12). At the 20th week, after confirmation of difference in body weight and plasma triglycerides, the animals were redistributed into 4 groups for the lycopene supplementation study: A control group (Co, *n* = 6), a control group supplemented with lycopene (Co+Ly, *n* = 6), an obese group (Ob, *n* = 6), and an obese group supplemented with lycopene (Ob+Ly, *n* = 6). The feed and water were ad libitum. The groups received lycopene solution (10 mg/kg/day) or vehicle (corn oil) via gavage for 10 weeks.

### 2.2. Diets

The diets used in this study were previously described by Francisqueti et al., (2017) [22]. Briefly, both diets were nutritionally balanced for micronutrients but different for macronutrients. The control diet was composed of soybean meal, sorghum, soybean peel, dextrin, soybean oil, vitamins, and minerals. The hypercaloric diet was designed to mimic the development of obesity associated with western dietary habits, being composed of soybean meal, sorghum, soybean peel, dextrin, sucrose, fructose, lard, vitamins and minerals, and addition of 25% sucrose in the drinking water. 

### 2.3. Lycopene Supplementation

The lycopene solution used in this study was the tomato oleoresin (Lyc-O-Mato^®^ 6% dewaxed) obtained from LycoRed Natural Products Industries, Beersheba, Israel. The solution was mixed with corn oil, checked for desired concentration absorbance (450 nm) and stored in a dark environment to ensure solution stability. The animals received lycopene solution (10 mg/kg/day) via gavage for 10 weeks [23,24]. The non-lycopene supplemented groups received corn oil vehicle (1 mL) via gavage.

### 2.4. Euthanasia

At the end of the 30-week period, after 8 h of fasting the animals were anesthetized (thiopental 120 mg/kg/i.p.) and euthanized by decapitation after the absence of foot reflex to obtain blood, liver, and adipose tissues (visceral, retroperitoneal and epididymal). Blood samples were collected in Falcon^®^ tubes (Kasvi, São José dos Pinhais, Paraná, Brazil) containing anticoagulant ethylenediamine tetraacetic acid (EDTA) and centrifuged at 2000× *g* and 4 °C for 10 min (Eppendorf^®^ Centrifuge 5804-R, Hamburg, Germany) and the plasma was stored in a freezer at −80 °C. The liver was fractionated in aliquots, the left hepatic lobe was stored in formaldehyde for histological analysis, and the remaining aliquots were stored in sterile cryotubes in a freezer at −80 °C. The fat deposits were dissected for weighing.

### 2.5. Nutritional and Obesity Characterization

The nutritional characterization was evaluated considering the chow intake, water intake, and caloric intake (diet + water). For that, water and chow were measured twice a week. The caloric intake was calculated by multiplying the daily chow intake by the energy value of each diet. The presence of obesity was established based on weight gain and adiposity index. For that, the body weight was measured weekly. The weight gain was calculated by subtracting the initial weight from the final weight of the animals [weight gain (g) = final weight (g) − initial weight (g)]. The adiposity index represents the ratio of the sum of the epididymal, visceral, and retroperitoneal fat deposits by the final weight multiplied by 100 [adiposity index (%) = (epididymal (g) + visceral (g) + retroperitoneal (g))/final weight (g) × 100].

### 2.6. Lycopene Bioavailability Evaluation

The presence of lycopene was determined in plasma and hepatic tissue homogenate. To extract the carotenoids, samples were incubated with internal standard (equinenone), chloroform/methanol CHCl_3_/CH_3_OH (3 mL, 2:1, *v*/*v*) and 500 mL of saline 8.5 g/L. Then the samples were centrifuged at 2000× *g* for 10 min and the supernatant was collected and hexane was added. The chloroform and hexane layers were evaporated under nitrogen and the residue was resuspended in 150 mL of ethanol and sonicated for 30 s. 50 μL of this aliquot was injected into the HPLC. The HPLC system was a Waters Alliance 2695 (Waters, Wilmington, MA, USA) and consisted of pump and chromatography bound to a 2996 programmable photodiode array detector, a C30 carotenoid column (3 mm, 150 × 3 × 4.6 mm, YMC, Wilmington, MA, USA) and Empower 3 chromatography data software (Milford, MA, USA). The HPLC system programmable photodiode array detector was set at 450 nm for carotenoids. The mobile phase consisted of ethanol/methanol/methyl-tert-butyl ether/water (83:15:2, *v*/*v*/*v*, 15 g/L with ammonium acetate in water, solvent A) and methanol/methyl-tert-butyl ether/water (8:90:2, *v*/*v*/*v*, 10 g/L with ammonium acetate in water, solvent B). The gradient procedure, at a flow rate of 1 mL/min (16 °C), was as follows: (1) 100% solvent A was used for 2 min followed by a 6 min linear gradient to 70% solvent A; (2) a 3 min hold followed by a 10 min linear gradient to 45% solvent A; (3) a 2 min hold, then a 10 min linear gradient to 5% solvent A; (4) a 4 min hold, then a 2 min linear gradient back to 100% solvent A. For the quantification of the chromatograms, a comparison was made between the area ratio of the substance and area of the internal standard obtained in the analysis [25].

### 2.7. Clinical Biochemistry

The glucose determination was performed by glycosimeter (Accu-Chek Performa; Roche Diagnostics Indianopolis, IN, USA). The concentrations of urea, creatinine, uric acid, total proteins, albumin, aspartate aminotransferase (AST), alanine aminotransferase (ALT), triglycerides, total cholesterol and its fractions, high-density lipoprotein cholesterol (HDL), and non-HDL cholesterol, were performed by an enzymatic colorimetric method with commercial kits (BioClin^®^, Belo Horizonte, MG, Brazil) in an automatic enzymatic analyzer system (Chemistry Analyzer BS-200, Mindray Medical International Limited, Shenzhen, China).

### 2.8. Hepatic Tissue Analysis

#### 2.8.1. Histology

Hepatic tissue was stored in 4% paraformaldehyde with 0.1 M phosphate buffer (pH 7.4) during the first 24 h. After, tissue was transferred to 70% ethyl alcohol until paraffin waxing. Histological sections obtained from the paraffin block were laid on slides and stained with hematoxylin and eosin (H&E). Macrovesicular steatosis and microvesicular steatosis were both separately scored and the severity was graded based on the percentage of the total area affected. The scores were: 0 (<5), 1 (≥5–33%), 2 (≥33–66%) and 3 (≥66%). The difference between macrovesicular and microvesicular steatosis was defined by whether the vacuoles displaced the nucleus to the side (macrovesicular) or not (microvesicular) [26]. Ten fields were analyzed per slide per animal. For the statistical analysis, it was considered the sum of the scores obtained in the 10 analyzed fields.

#### 2.8.2. Total Proteins

The tissue samples were homogenized in phosphate-buffered saline (PBS) at a ratio of 1:10 (sample:PBS). Total protein concentrations in hepatic tissue were measured in the homogenate by a colorimetric method using the biuret reagent (BioClin, Quibasa Química Básica Ltd.a., Belo Horizonte, Minas Gerais, Brazil) and were verified in an automatic enzymatic analyzer system (Chemistry Analyzer BS-200, Mindray Medical International Ltd., Shenzhen, China). The results were used to correct the parameters of the inflammation and redox state.

#### 2.8.3. Inflammatory Biomarkers

The hepatic concentrations of interleukin-6 (IL-6) and tumor necrosis factor-α (TNF-α) were quantified in the tissue homogenate by enzyme-linked immunosorbent assay (ELISA) using specific commercial kits (R&D Systems^®^, Minneapolis, MN, USA) and a micro-plate reader (Spectra MAX 190, Molecular Devices, Sunny Valley, CA, USA). The results were corrected for the total protein present in the tissue and expressed in picogram per milligram of protein (pg/mg protein). The tissue samples were homogenized in phosphate-buffered saline (PBS) at the ratio of 1:10 (sample:PBS).

#### 2.8.4. Redox State

##### Oxidative Damage to Lipids

The malondialdehyde (MDA) concentration was obtained by high performance liquid chromatography with fluorometric detection (HPLC, system LC10A, Shimadzu, Japan). The hepatic tissue homogenate (100 µL) was incubated with thiobarbituric acid (TBA 42 mmol/L, 200 μL) and 1% ortho-phosphoric acid (700 μL) at 100 °C for 60 min to form the fluorescent adduct TBA-MDA. Deproteinization was performed with 2 mmol/L sodium hydroxide and methanol (NaOH:MetOH; 1:1) submitted to centrifugation (2000× *g*, 5 min). After 50 μL of the supernatant was filtered and injected into an octadecylsilic column (ODS-2, 150 × 4.6 mm, 5 µm; Spherisarb^®^, Waters). The isocratic mobile phase comprised of the phosphate buffer (10 mmol/L, pH:6.8) and HPLC grade methanol (60:40 *v/v*), it was run through the system at a flow rate of 0.5 mL/min. The calibration curve was obtained with the preparation of tetra-ethoxy propane solutions (TEP). Fluorimetric detection was performed at 527 nm of excitation and 551 nm of emission. The results were expressed as nanomol per milligram protein (nmol/mg protein) [27].

##### Antioxidant Enzyme Activity

In these analyzes, the activity of superoxide dismutase (SOD) and catalase (CAT) was evaluated. Therefore, 100mg liver was homogenized (1:20 *v*/*v*) in KH_2_PO_4_ (10 mmol/L)/KCl (120 mmol/L), pH 7.4, and centrifuged at 2.000× *g* for 20 min. SOD activity was measured based on the inhibition of a superoxide radical reaction with pyrogallol, and the absorbance values were measured at 420 nm [28]. The values were expressed as units per milligram of protein (U/mg protein). CAT activity was evaluated by following the decrease in the levels of hydrogen peroxide, absorbance was measured at 240 nm [29]. The results were expressed as picomol per milligram of protein (pmol/mg protein).

##### Hepatic Antioxidant Capacity

The hepatic tissue antioxidant capacity was determined by the total antioxidant performance test (TAP) using a VICTOR X2 reader (Perkin Elmer-Boston, MA, USA) [30]. This assay utilized 100 µL of homogenate, which was incubated with the fluorescent indicator BODIPY (4,4-difluoro-4-bora-3a, 4a-diaza-s-indacene) for 10 min at 37 °C. Then the free radical generator 2,2’Azobis(2-amidino-propane)-dihydrochloride (AAPH) was added to the samples. The prepared samples were applied in triplicates, 200 µL in each well on specific plates. Phosphatidylcholine (PC) was used as a hydrophilic matrix reference. The fluorescence reader (Wallac Vitor 2X^®^, Perkin-Elmer, Boston, MA, USA) was programmed to perform readings every 5 min for 3 h and 30 min. The analyses were performed in triplicate, and the results represented the percent protection.

### 2.9. Statistical Analysis

Data were presented as means ± standard deviation (SD) or median (interquartile range). Differences among the groups were determined by two-way ANOVA with Holm-Sidak post-hoc test or by Kruskall Wallis test with Tukey post-hoc test. The comparison between the 2 groups was performed by the *T*-test. These statistical analyses were performed using the software Sigma Stat for Windows Version 3.5 (Systat Software Inc., San Jose, CA, USA). For the histological parameters, the Poasson distribution or the binomial distribution followed by the post-hoc test Wald multi-comparison were used. These statistical analyses were performed by an experienced statistician using the software Statistical Analysis System (SAS) 9.4 (SAS Institute Inc., Campus Drive Cary, NC, USA). A *p* value of ≤0.05 was considered statistically significant.

## 3. Results

### 3.1. Lycopene Does Not Influence Nutritional and Adiposity Markers

Both Ob and Ob+Ly groups presented lower chow intake. There was no difference between the groups in water and caloric intake. There was no difference between the initial weights. The obese groups (Ob and Ob+Ly) presented a higher final weight and weight gain (Table 1).

**Figure 1:**
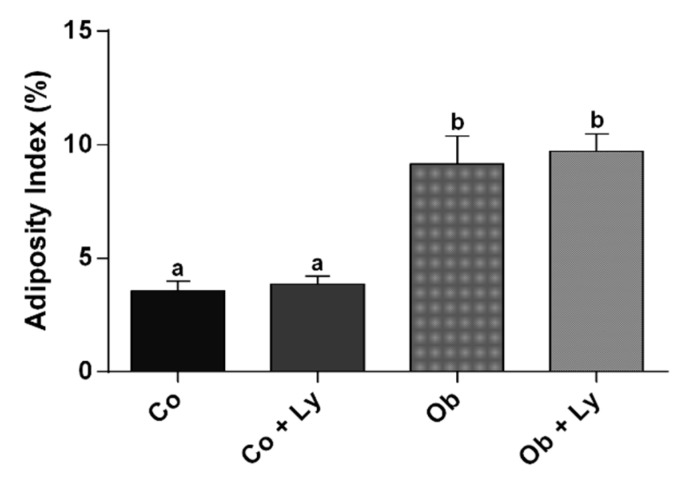
Determination of the presence of obesity: Adiposity index (%). Co: Control group; Co+Ly: control group supplemented with lycopene; Ob: Obese group; Ob+Ly: Hypercaloric group supplemented with lycopene. Data expressed in mean ± standard deviation. Comparison by two-way ANOVA with Holm-Sidak post-hoc test. Different letters correspond to the significant statistical difference (*p* <0.05); *n* = 6 animals/group.

The determination of the presence of obesity is shown in Figure 1. Similar to weight gain, the adiposity index was higher in the groups Ob and Ob+Ly.

### 3.2. Lycopene Is Available in Plasma and Liver of Supplemented Rats

The lycopene bioavailability is shown in Table 2. It is possible to verify the presence of lycopene in both groups, which were supplemented (Co+Ly and Ob+Ly).

### 3.3. Lycopene Influences Plasma Lipid Markers

Table 3 presents the biochemical analysis related to renal and hepatic functions. There was no difference between the groups for all the parameters presented in Table 3.

Markers for lipid and glucose metabolism are exhibited in Table 4. The fasting blood glucose was higher in the obese groups (Ob and Ob+Ly) independent of lycopene supplementation. Plasma triglycerides were higher only in the Ob group. Similarly, the Ob group presented higher hepatic triglycerides levels, and the Ob+Ly group showed similar levels to the control groups (Co and Co+Ly). The total cholesterol was higher in the groups supplemented with lycopene. This finding could be influenced by increasing HDL cholesterol levels also observed in supplemented groups, mainly the Ob+Ly group. There was no difference between groups for non-HDL cholesterol (Table 4).

### 3.4. Lycopene Ameliorates Obesity-Related Hepatic Steatosis

**Figure 2:**
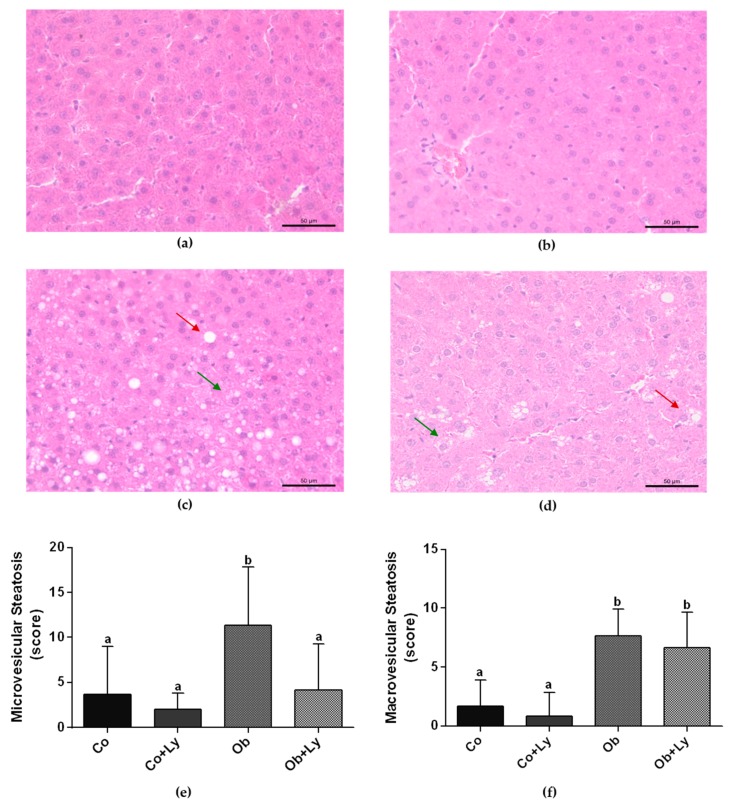
Determination of the hepatic steatosis by hepatic tissue histology stained with haematoxylin and eosin (H&E) (**a**) Illustrative picture (40× magnification) of the control group (Co); (**b**) illustrative picture (40× magnification) of the control group supplemented with lycopene group (Co+Ly); (**c**) illustrative picture (40× magnification) of the obese group (Ob); (**d**) illustrative picture (40× magnification) of the obese group supplemented with lycopene (Ob+Ly); (**e**) microvesicular steatosis (score); (**f**) macrovesicular steatosis (score). Presence of macro (red arrows) and microvesicular (green arrow) steatosis in obese groups. Data expressed in mean ± standard deviation. Comparison by Poasson distribution followed by the post-hoc test Wald multi-comparison. Different letters correspond to the significant statistical difference (*p* < 0.05); *n* = 6 animals/group.

Figure 2 shows the determination of the hepatic steatosis by histological parameters. Independent of lycopene supplementation, the hepatic tissue of the obese animals (Ob and Ob+Ly) presented higher macrovesicular steatosis. Lycopene was efficient in attenuating the microvesicular steatosis of treated obese animals (Ob+Ly).

### 3.5. Lycopene Ameliorates TNF-α Levels in Hepatic Tissue

**Figure 3:**
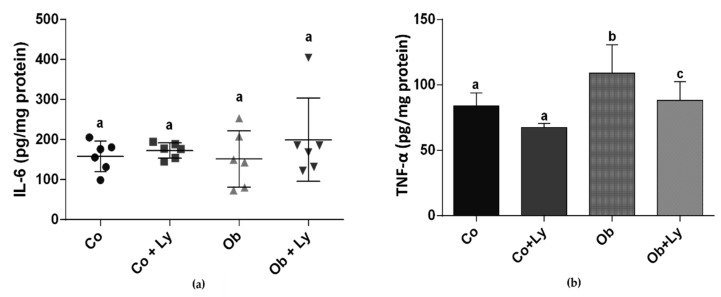
Inflammatory process in hepatic tissue: (**a**) IL-6 (pg/mg protein); (**b**) TNF-α (pg/mg protein). Co: Control group; Co+Ly: Control group supplemented with lycopene; Ob: Obese group; Ob+Ly: Hypercaloric group supplemented with lycopene. Data parametric expressed in mean ± standard deviation. Comparison by two-way ANOVA with Holm-Sidak post-hoc test. Data non-parametric expressed in median and interquartile range. Comparison by Kruskall Wallis test with Tukey *post-hoc* test. Different letters correspond to the significant statistical difference (*p* < 0.05); *n* = 6 animals/group. IL-6: Interleukin-6; TNF-α: Tumor necrosis factor-α.

In hepatic tissue, there was no difference between groups for the pro-inflammatory cytokine IL-6. The Ob group presented the highest levels of TNF-α and the supplementation with lycopene in obese animals (Ob+Ly) was effective in the attenuation of cytokine levels (Figure 3).

### 3.6. Lycopene Shows Anti-Lipid Peroxidation Activity in Hepatic Tissue

**Figure 4:**
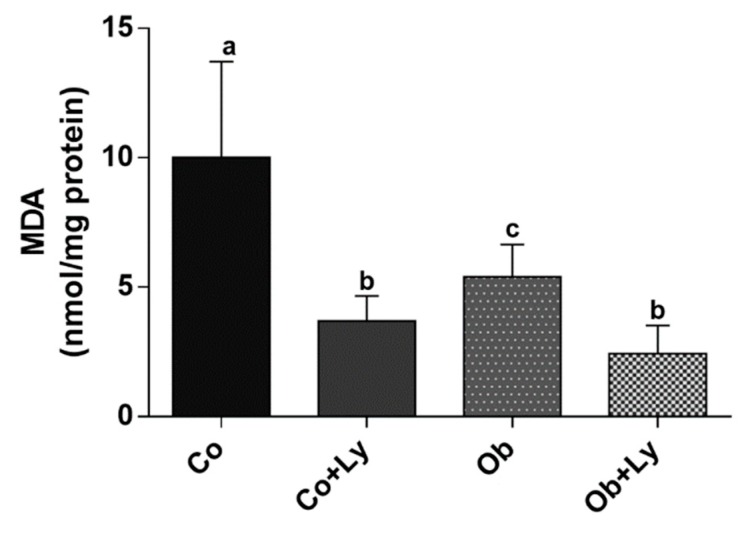
Assessment of lipid peroxidation through the quantification of malondialdehyde (MDA) in the liver: MDA (nmol/mg protein). Co: Control group; Co+Ly: Control group supplemented with lycopene; Ob: Obese group; Ob+Ly: Hypercaloric group supplemented with lycopene. Data parametric expressed in mean ± standard deviation. Comparison by two-way ANOVA with Holm-Sidak post-hoc. Different letters correspond to the significant statistical difference (*p* < 0.05); *n* = 6 animals/group.

The lipid peroxidation represented by MDA formation in the liver was higher in the control group. Lycopene was effective in diminished oxidative damage to lipids since it is possible to observe the lower levels of MDA in supplemented groups (Co+Ly and Ob+Ly) (Figure 4).

### 3.7. Lycopene Improves Antioxidant Enzyme Activity in Hepatic Tissue

**Figure 5:**
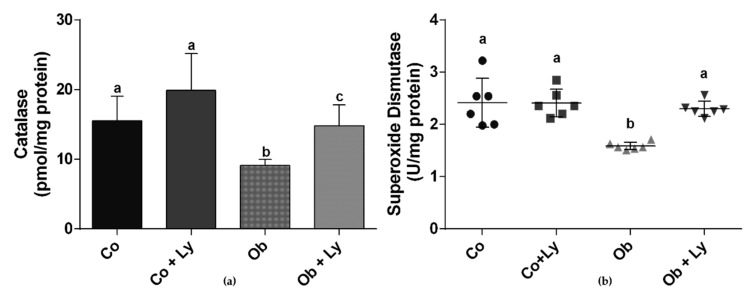
Antioxidant enzyme activity: (**a**) Catalase (pmol/mg protein); (**b**) Superoxide Dismutase (U/mg protein). Co: Control group; Co+Ly: Control group supplemented with lycopene; Ob: Obese group; Ob+Ly: Hypercaloric group supplemented with lycopene. Data parametric expressed in mean ± standard deviation. Comparison by two-way ANOVA with Holm-Sidak post-hoc test. Data non-parametric expressed in median and interquartile range. Comparison by Kruskall Wallis test with Tukey post-hoc test. Different letters correspond to the significant statistical difference (*p* < 0.05); *n* = 6 animals/group.

Figure 5 shows the antioxidant enzyme activity in the liver. Obese groups (Ob and Ob+Ly) showed reduced activity of CAT and the lycopene supplementation (Ob+Ly) improved CAT activity. The lycopene also had a positive effect on the SOD activity of the Ob+Ly group, which reached similar activity to the control groups (Co and Co+Ly).

### 3.8. Lycopene Improves Total Antioxidant Capacity in Hepatic Tissue of Obese Rats

**Figure 6:**
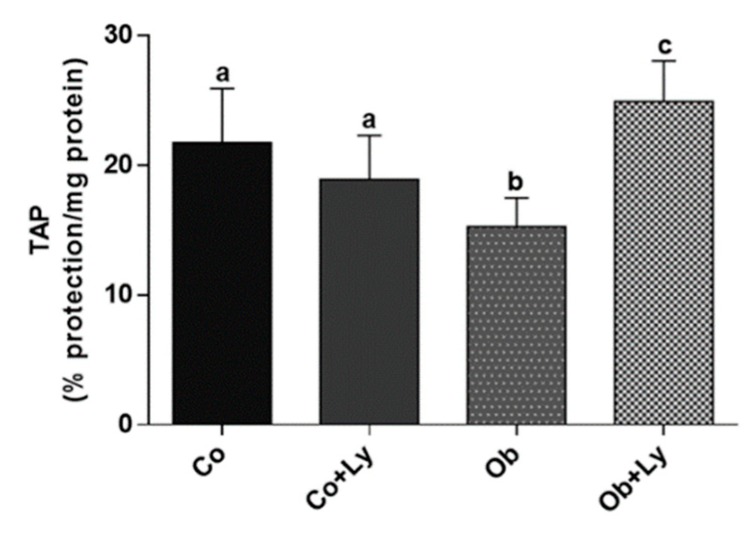
Total antioxidant performance test (TAP) (% protection/mg protein). Co: Control group; Co+Ly: Control group supplemented with lycopene; Ob: Obese group; Ob+Ly: Hypercaloric group supplemented with lycopene. Data parametric expressed in mean ± standard deviation. Comparison by two-way ANOVA with Holm-Sidak post-hoc. Different letters correspond to the significant statistical difference (*p* < 0.05); *n* = 6 animals/group.

Figure 6 shows the total hepatic antioxidant capacity. The antioxidant protection was higher in the obese group supplemented with lycopene (Ob+Ly).

## 4. Discussion

The aim of this study was to investigate the therapeutic potential of lycopene on the pathophysiological processes of NAFLD in obese animals fed a hypercaloric diet. The experimental model proposed in this study promoted metabolic and hepatic changes, represented by increased levels of fasting blood glucose and of plasmatic and hepatic triglycerides both associated with the accumulation of fat in the liver. This hepatic disturbance could be observed in the histological analysis through macro and microvesicular steatosis. However, there were no changes in liver enzymes ALT and AST evidencing that there was no progression of the disease. Although the model did not transpose steatosis, the obese animals (Ob) presented high levels of TNF-α levels, a potent pro-inflammatory cytokine. A greater weight gain and a different body composition represented by the adiposity index was also observed. The animals, which were fed a hypercaloric diet, ate a smaller amount of chow in grams. This event did not interfere with the caloric intake value as these animals received a higher caloric amount derived from water intake containing 25% sucrose. Despite the similar caloric intake between the groups, the difference in diet composition, in other words, its macronutrient quality and distribution reflected a greater weight gain in the groups which received the hypercaloric diet, in addition to all metabolic changes remarked. 

Supplementation with lycopene did not influence the chow intake behavior and the weight gain of the treated groups. Although several studies have listed lycopene as an inducer of adiponectin concentrations [23,31], and adipokine, where serum levels are negatively associated with obesity by increasing energy expenditure [32], few have signed lycopene as a subsidiary in weight control [31]. The lycopene also did not influence the fasting blood glucose, although the action of lycopene in glycemic control of diabetic animals was found in the literature [33,34]. 

Lycopene is absorbed in the intestine and stored in addition to other organs in the liver where it exerts its biological activities [35]. Thus, the lycopene acts as a protective factor in the organ due to its antioxidant effect [36]. However, although redox imbalance state plays an important role in the pathogenesis of NAFLD, other metabolic disturbances are important as changes in energy metabolism [35]. Accordingly, the positive effects of lycopene were not only restricted to the protective effect in the antioxidant hepatic system, discussed later, one of the most relevant effects of lycopene supplementation was its action on lipid metabolism.

The liver is responsible for several functions in the body, such as cholesterol, fatty acids, carbohydrates, and protein metabolism, besides the formation of plasma proteins, and production of bile among other processes [37]. Some members of the nuclear receptor (NR) family are responsible for regulating these mechanisms in order to maintain the hepatic and body homeostasis. These receptors form heterodimers with the retinoid X receptor (RXR), resulting in gene transcription [38]. The heterodimers may be activated by specific ligands for each component of the pair or by simultaneous binding. Lycopene seems to act as an RXR ligand and is, therefore, capable of activating several NRs, although it has an important affinity for peroxisome proliferator-activated receptors (PPARs) family [39]. Anyway, these receptors are highly expressed in the liver and are involved in the transcription of enzymes related to lipid metabolism. Although all have important metabolic functions the PPAR-α is identified as the master regulator of hepatic lipid metabolism [40]. Its expression is inversely proportional to the progression of the histological severity of NAFLD and directly proportional to the improvement of the inflammatory state [41]. PPAR-α plays a key role in lipid catabolism through regulating target genes involved in β-oxidation, uptake and activation of fatty acids, and lipolysis [42]. Thus, PPAR-α has been targeted in the development of drugs for the NAFLD treatment [43], and lycopene was shown to have a protective effect on the disease through its binding in these NR [44].

Histologically, NAFLD is characterized by macrovesicular steatosis, that is, large or small lipid droplets are present in the cytoplasm with nucleus displacement [45]. However, approximately 10% of the liver biopsies of patients with NALFD present microvesicular steatosis [46], which is characterized by the accumulation of innumerable lipid droplets with a centrally placed nucleus [47]. The extensive microvesicular steatosis is associated with alcoholic fatty liver disease [48]. The difference between macrovesicular and microvesicular steatosis is not restricted to the histological aspect. Macrovesicular steatosis when alone is associated with a good prognosis with rare progression to fibrosis or cirrhosis [46]. Contrary, the microvesicular steatosis is a serious condition related to fibrosis, cholestasis, necrosis [49], and moreover to an impairment of the mitochondrial fatty acid oxidation [50]. The details surrounding the formation of these lipid droplets remain to be defined [51], and this study is not able to discern the relationship between micro and macrovesicular steatosis.

Interestingly, in the present study, lycopene was able to reduce microvesicular steatosis. This effect could be attributed to enhanced β-oxidation, an impaired pathway in this pattern of lipid accumulation [50]. Gala Martín-Pozuelo and colleagues [52] followed for seven weeks 24 Sprague-Dawley rats fed a hypercaloric diet and provided with water or tomato juice. The researchers concluded that the lycopene increased the activity of some enzymes involved in β-oxidation in the liver of animals that received the juice. This event was attributed to the activation of PPAR-α by lycopene.

Several researchers with animal models have investigated the therapeutic effect of lycopene on blood lipids [53,54,55,56]. In this study, an increase in total cholesterol was observed in the groups supplemented with lycopene. Such a finding may be justified by the higher levels of HDL in these animals since there was no change in non-HDL levels. HDL is the lipoprotein responsible for the reverse transport of cholesterol by removing cholesterol from the peripheral vessels and directing it to the liver. This added to other properties such as antioxidative, anti-inflammatory, vasodilatory, antithrombotic, and cytoprotective, which makes HDL a protective factor for cardiovascular diseases intrinsically associated with obesity [57]. In this sense, pharmacological and dietary therapies have been investigated with the objective of elevating HDL [58]. This lipoprotein beyond cholesterol is responsible for the transport of other molecules, including proteins, small RNAs, bioactive lipids, hormones, vitamins, and carotenoids. Hence, it has been suggested that the content of carotenoids plays a role in protecting HDL from oxidative modification [59].

Among the reasons for high HDL levels is the increase in adipose tissue observed in obesity, which is characterized by a chronic inflammation state of low grade. Serum amyloid A (SAA) is one of these pro-inflammatory proteins, which in high concentrations displaces apoprotein AI (apoA-I) as the predominantly apolipoprotein of HDL, making the lipoprotein dysfunctional [60]. McEneny and colleagues [61] studied the effects of lycopene on HDL and SAA in moderately overweight middle-aged men and women. Lycopene intervention diminished inflammation by decreasing systemic levels of SAA, hence reducing the association of SAA with HDL, and it even positively influenced the structural and/or functional composition of the apo AI. In this way, the carotenoid helped to restore the HDL functions. In addition, HDL is produced in the liver and intestine and its formation is controlled by NR [62]. PPAR-α is able to increase the synthesis of apoAI and the ATP-binding cassette A1 transporter (ABCA1) expression. In the intestine, activation of PPAR-α is facilitated by the binding of agonists present in the diet, such as lycopene. The action of lycopene in both the liver and the intestine results in increased plasma HDL levels [63]. Further, the agonist effect of lycopene on LXR also seems to increase the expression of ABCA1 and hence increase HDL levels [64].

The inflammation and oxidative stress are two relevant features of NAFLD. The liver inflammatory process represents an important pathway in the development of NAFLD/NASH, therefore, research has sought its control [65]. The hypercaloric diet increases the hepatic levels of the pro-inflammatory cytokine TNF-α, probably through activation of nuclear factor kappa B (NF-κB) signaling [66]. It is established in the literature that the anti-inflammatory potential of lycopene by promoting a significant inhibition of the NF-κB is probably mediated by PPARs [67]. In the present study, lycopene reduced the levels of TNF-α, reflecting control of the pro-inflammatory mechanisms, and also contributing to a reduction in pattern and extent of liver damage [68]. The inflammatory response may promote ROS production by the immune system cells, which contributes to liver damage [69]. Furthermore, ROS are able to activate NF-κB to induce pro-inflammatory cytokines contributing to this vicious cycle between inflammation and oxidative stress [14]. 

The accumulation of hepatic lipid related to a hypercaloric diet induces a metabolic shift in order to overcome this amount of lipid. This shift includes improvements in β-oxidation, Krebs cycle, and stimulation of oxidative phosphorylation (OXPHOS) [70]. However, with the chronic overnutrition, the mitochondrial adaptation is insufficient, leading to ROS overproduction [14]. One of the consequences of the redox imbalance is the lipid lipoperoxidation represented here by the formation of MDA. The MDA levels were higher in the control groups due to the lipid source of diet since the polyunsaturated fatty acids (PUFAs) present in soybean oil are more susceptible to lipid peroxidation [71]. Nevertheless, lycopene supplementation elevated SOD and CAT activity and significantly decreased the MDA level in hepatic tissue, probably due to nuclear factor erythroid 2–related factor 2 (Nrf2) activation. The Nrf2 system plays a crucial role in the regulation of redox homeostasis by orchestrating the antioxidant defense mechanisms by inducing endogenous antioxidant enzymes [72]. In addition, lycopene was efficient in increasing total antioxidant capacity in the obese group supplemented with lycopene (Ob+Ly), contributing to the control of pro-oxidative and pro-inflammatory processes.

Despite the outcome of lycopene effects on NAFLD observed in this study, there are some limitations regarding the translation of this study to humans. The lycopene concentration used in the treatment is higher than that could be offered by a balanced diet. More studies are needed to determine the lycopene effects on NAFLD in humans as well as the appropriated dose for treatment.

## 5. Conclusions

**Figure 7:**
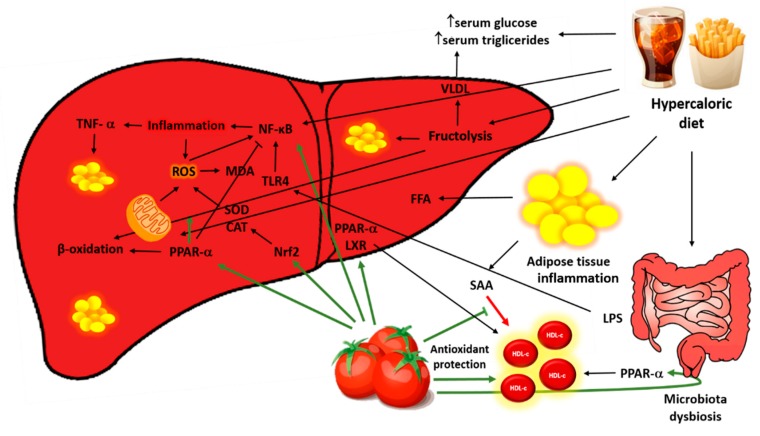
Role of lycopene in the pathophysiological process of non-alcoholic fatty liver disease (NAFLD). The hypercaloric diet provides large amounts of fat and especially sugars, which saturate the liver’s ability to export triglycerides leading to its accumulation in the hepatocyte in addition to increased levels of triglyceride and plasma glucose. These macronutrients also activate inflammatory pathways directly by NF-κB activation and indirectly through alteration of intestinal permeability and translocation of LPS to the liver. The excess nutrients overload the oxidation capacity of mitochondria leading to ROS production, establishing a mutually dependent process between inflammation and oxidative stress. Lycopene was efficient in controlling the inflammatory and oxidative pathways, as well as in the antioxidant activity and assisted in the control of the accumulation of hepatic lipids, activating β-oxidation. Furthermore, the carotenoid improved blood lipids profile by lowering triglycerides and increasing HDL-C levels. LPS: Lipopolysaccharides; SAA: Serum amyloid A; HDL-c: High-density lipoprotein cholesterol; VLDL: Very low-density lipoprotein, FFA: Free fatty acid; Nrf2: Nuclear factor erythroid 2–related factor 2; PPAR-α: Peroxisome proliferator-activated receptor α; SOD: Superoxide dismutase CAT: Catalase; TLR4: Toll-like receptor 4; ROS: Reactive oxygen species; MDA: Malondialdehyde; TNF-α: Tumor necrosis factor-α.

In summary, lycopene decreases plasmatic and hepatic triglycerides levels, increased HDL-c, mitigates microvesicular steatosis, attenuates levels of the pro-inflammatory cytokine, and contributes to the redox balance in the liver (Figure 7) In view of these results, it may be concluded that lycopene modulates important pathophysiological processes of the NAFLD, being a therapeutic potential for disease control.

## Figures and Tables

**Table 1:** Intake and weight characteristics.

Parameters	Co	Co+Ly	Ob	Ob+Ly
Chow intake (g/d)	24 ± 0.8 ^a^	24.4 ± 0.4 ^a^	12.8 ± 0.6 ^b^	11.8 ± 0.4 ^b^
Water intake (mL/d)	24.1 ± 0.6 ^a^	27 ± 1.4 ^a^	23.9 ± 0.6 ^a^	23.7 ± 0.4 ^a^
Caloric intake (kcal/d)	89.9 ± 2.9 ^a^	88.9 ± 0.9 ^a^	95 ± 3.9 ^a^	94.2 ± 1.8 ^a^
Initial Weight (g)	192 ± 28.4 ^a^	209 ± 14.3 ^a^	212 ± 21.4 ^a^	198 ± 11.6 ^a^
Final Weight (g)	490 ± 43.7 ^a^	470 ± 59.6 ^a^	568.5 ± 75.2 ^b^	552 ± 42.5 ^b^
Weight Gain (g)	297 ± 33.7 ^a^	260 ± 52.0 ^a^	356 ± 72.9 ^b^	354 ± 34.2 ^b^

Co: Control group; Co+Ly: Control group supplemented with lycopene; Ob: Obese group; Ob+Ly: hypercaloric group supplemented with lycopene. Data parametric expressed in mean ± standard deviation. Comparison by two-way ANOVA with Holm Sidak post-hoc test. Different letters correspond to the significant statistical difference (*p* <0.05); *n* = 6 animals/group.

**Table 2:** Lycopene concentration in hepatic tissue and plasma.

Parameters	Co	Co+Ly	Ob	Ob+Ly
Liver (µg/100 g tissue)	ND	47.32 ± 5.95 ^a^	ND	25 ± 2.91 ^b^
Plasma (µg/mL)	ND	3.18 ± 0.586 ^a^	ND	5.22 ± 2.31 ^a^

Co: Control group; Co+Ly: Control group supplemented with lycopene; Ob: Obese group; Ob+Ly: Hypercaloric group supplemented with lycopene. ND: Not detectable. Data parametric expressed in mean ± standard deviation. Comparison by t-test. Different letters correspond to the significant statistical difference (*p* < 0.05); *n* = 6 animals/group.

**Table 3:** Markers for clinical biochemistry.

Parameters	Co	Co+Ly	Ob	Ob+Ly
Urea (mg/dL)	54.2 ± 8.8 ^a^	51.5 ± 3.9 ^a^	48.0 ± 33.6 ^a^	42.8 ± 20.9 ^a^
Creatinine (mg/dL)	0.432 ± 0.034 ^a^	0.433 ± 0.057 ^a^	0.568 ± 0.291 ^a^	0.504 ± 0.092 ^a^
Uric Acid (mg/dL)	0.544 ± 0.103 ^a^	0.693 ± 0.307 ^a^	0.695 ± 0.122 ^a^	0.800 ± 0.166 ^a^
AST (U/L)	149 (100–230) ^a^	123 (113–152) ^a^	112 (87–213) ^a^	141 (135–179) ^a^
ALT (U/L)	44.5 (38.7–161) ^a^	50.5 (35.5–106) ^a^	51 (32.7–133) ^a^	38.5 (32.5–95.7) ^a^
Albumin (g/dL)	2.7 ± 0.1 ^a^	2.6 ± 0.1 ^a^	2.7 ± 0.1 ^a^	2.7 ± 0.1 ^a^
Total Proteins (g/dL)	5.6 ± 0.1 ^a^	5.6 ± 0.3 ^a^	5.9 ± 0.2 ^a^	5.9 ± 0.3 ^a^

Co: Control group; Co+Ly: Control group supplemented with lycopene; Ob: Obese group; Ob+Ly: Hypercaloric group supplemented with lycopene. AST: Aspartate aminotransferase; ALT: Alanine aminotransferase. Data parametric expressed in mean ± standard deviation. Comparison by two-way ANOVA with Holm Sidak *post-hoc* test. Data non-parametric expressed in median and interquartile range. Comparison by Kruskall Wallis test with Tukey post-hoc test. Different letters correspond to the significant statistical difference (*p* < 0.05); *n* = 6 animals/group.

**Table 4:** Markers for lipid and glucose metabolism.

Parameters	Co	Co+Ly	Ob	Ob+Ly
Fasting Blood Glucose (mg/dL)	74.2 ± 7.56 ^a^	91.6 ± 16.6 ^a^	102 ± 21.4 ^b^	104 ± 7.50 ^b^
Triglycerides (mg/dL)	62.0 ± 20.3 ^a^	77.3 ± 32.5 ^a^	113 ± 41.8 ^b^	93.9 ± 12.4 ^a^
Hepatic Triglycerides (mg/dL)	20.6 ± 4.29 ^a^	20.6 ± 2.92 ^a^	33.2 ± 7.9 ^b^	29.3 ± 5.59 ^a,b^
Total Cholesterol (mg/dL)	51.5 ± 8.80 ^a^	60.0 ± 12.1 ^a. b^	56.9 ± 14.1 ^a^	71.7 ± 10.8 ^b^
HDL Cholesterol (mg/dL)	18.0 ± 3.25 ^a^	23.5 ± 3.94 ^b^	19.9 ± 5.43 ^a^	29.0 ± 2.69 ^c^
Non-HDL Cholesterol (mg/dL)	33.5 ± 5.95 ^a^	36.4 ± 9.09 ^a^	37.0 ± 9.86 ^a^	42.6 ± 8.87 ^a^

Co: Control group; Co+Ly: Control group supplemented with lycopene; Ob: Obese group; Ob+Ly: Hypercaloric group supplemented with lycopene. HDL: high density lipoprotein. Data parametric expressed in mean ± standard deviation. Comparison by two-way ANOVA with Holm-Sidak post-hoc test. Different letters correspond to the significant statistical difference (*p* <0.05); *n* = 6 animals/group.
